# International Medical Congress

**Published:** 1867-12

**Authors:** 


### CHICAGO MEDICAL JOURNAL.

Vol. XXIV. DECEMBER, 1867. No. 12.

### Original Contributions.

### INTERNATIONAL MEDICAL CONGRESS,

Convened at Paris, Aug. 16th, 1867, at 2 o'clock P.M.

The Medical Congress opened amidst a compact crowd of physicians, French and foreign. The grand amphitheatre of l'Ecole de Medicin was completely filled, its capacity being 1000. The opening address of the President, M. Bouillaud, full of souvenirs of the past and hopes for the future, was heartily received. We give a few extracts:

"International Congresses would certainly be able to become very efficacious in furthering the progress of science, and in binding the profession in fraternal bonds, if they could be able to enter into the manners and habitudes of the different peoples. Our neighbors across the sea, more habituated than we to reunions, perhaps understand better the art of feasting their guests; but, in all things, an apprenticeship is necessary."

"The utility of International Congresses should be confirmed by the results, and not admitted without question. Until then, a great many will hesitate, and will not bring in their contingent of knowledge and good will; thereby diminishing the time and chances of success."

"Medical Congresses will not be able to offer all their advantages, until they are constituted as grand assizes; where all new ideas and all questions will be debated without inutile phraseology, by all those who wish to labor actively in the progress of science."

"It is evidently necessary that all the facts in appearance contradictory, all those that throw doubt upon the subject under discussion, may be exposed by the observers themselves, and in presence of one another. This cannot be done in an academy or in a society of limited numbers, but it is possible in a world's congress of enlightened, disingenuous, scientific men."

"We hope, then, for more international congresses in the future—grand assemblages which may be able to vivify the intelligences, reverse the systems of hazard, and make known to each of the peoples the doctrine of its neighbors."

After his allocution, M. Bouillaud was nominated by acclamation for Permanent President of the Congress, and the bureau was completed by the choice of the following:

*Foreign Vice-Presidents.*—Profs. Virchow, Berlin; Hallo, Prague; Lambi, Karkoff; Meric, London; Palasciano, Naples; Wleminckz, Bruxelles.

*French Vice-Presidents.*—Prof. Berare, Montpelier; Prof. Gintrac, Bordeaux; Prof. Baron Larrey, Paris; Dr. Ricord, Paris; Prof. Roux, Toulon; Prof. Tessier, Lyons.

*Secretary-General.*—Dr. Jaccoud.

*Treasurer.*—Dr. Vidal.

FIRST QUESTION.—*Anatomy, Pathology, and Physiology of Tubercle.*

M. Villemin, Val du Grace, prefaced his paper with the remark, that at first he took part with the school of Virchow, in regarding only as true tuberculosis the gray granulations, semi-transparent, etc., and distinguished from this, what this school has named pneumonia caseous. But now he viewed all these alterations as tuberculosis of the same nature.

Here is an extract from his paper on the subject:—One can represent a tubercular node by three concentric zones, corresponding to three different degrees in the evolution of the elements which concur in its formation. One external zone, where cells are seen already more voluminous than those of normal tissue, and in which appears many nuclei; a middle zone, represented by element of various dimensions, more or less compressed, one against the other, and containing a variable number of nuclei—this latter is the "proliferant zone;" finally, a central zone, where are found accumulated nuclei and little cells, similar to globules of pus in inflammation, the product final of the multiplication of elements, notwithstanding, one not rarely meets with cells of larger dimensions in this situation.

The retrogressive metamorphosis of tubercle presents itself under various aspects. In certain cases, it assumes the condition of fatty granulations, very small, and relatively rare, reflecting light brilliantly, and having a shriveled and dry appearance. It is a sort of mummyfication, according to Mr. Kuss. At other times, the fatty globules are more voluminous and more abundant. We think that these two forms of retrogressive transformation tends towards two different terminations. The first seems to terminate with greater facility in cretification, and the second in softening.

Now and then, one may observe granulations which contain hardly any elements of small dimensions at their centre, and which are composed of cells of variable size, in the way of active multiplication, and which are destroyed by retrograde metamorphoses before the proliferation may have arrived at its final term. A section of a nodosity of this species seems to be constituted of elements similar to those spoken of as constituting the middle zone. There is a predominance of voluminous cells upon the nuclei and small cells, whilst, on the contrary, in the granulation type, the elements of large dimensions are rare in the central part, and even rare in the middle zone.

In the granulation type, necrobiosis, also, sometimes attacks the large cells in the way of proliferation, which tends to the obliteration of the vessels by the encroachment of the nodosities one upon the other. These cellules, thus deprived of nutriment, die; and such is, without doubt, the cause of the rapid march of certain cases of phthisis.

In tuberculization of the lungs, one encounters many granulations which have their seat in the connective tissue of the inter-lobular substance, but the greatest number are found in the vesicles themselves which are found filled with cells in the way of multiplication. In this instance, we have considered these contents of the vesicles as derived from the epithelium of the lining membrane, and distinct from tubercular granulations, but we have abandoned this interpretation. We are now assured that the partitions which separate pulmonary vesicles are not homogeneous, but that they contain in their thickness a proper cellular element which is common to connective tissue which may be the seat of granulation, as in other situations, for example, interlobular tissue. Respecting the existence of an epithelium on the surfaces of the pulmonary vesicles, we consider it problematical.

In the lungs, tubercular granulations present, as elsewhere, the largest cells towards the circumference, they are there even sometimes very large, containing, sometimes, ten or fifteen nuclei, which proliferate in their turn. The areolæ are filled by this proliferation, and often the necrobiosis seizes at one time all the elements, not only those which are small and form the centre of the focus, but those found at the circumference and in a state of proliferation.

This happens always, when the foci are closely aggregated one against the other. Then a section of the part exposes to view only proliferant cells filling more or less completely the areolæ. It is to this form that has been given the name of caseous pneumonia, tuberculous, epithelial, disseminated, chronic, etc., etc.

But these elements proceed, manifestly, from the cell nuclei which are found in the partitions separating the lung vesicles. In tubercles proceeding from serous and mucous membranes, from lymphatic ganglia, the proliferant zone is composed of cells absolutely identical in form, dimensions, and all other characters, with the above. It is but by compression, the one against the other, that they sometimes show plain faces and appear like epithelium, they are never soldered together. Besides, in a connective tissue, the tumefaction and cellular proliferation does not differ from that observed in inflammation. It is only by the final result that the nature of the process can be judged. Inflammation ends in pus or induration; tubercle, in fatty metamorphosis, or in hasty necrobiosis, as in that which is named caseous pneumonia.

If, in this case, one had to do with an inflammatory product simply, and of an epithelial nature throughout, we would not have a suppression of the circulation affected, and the lung, instead of taking on in the end an anæmic aspect and a dry consistence, would be remarkable, on the contrary, for the turgescence and engorgement which characterizes the inflammatory process. (M. Villemin recalled the resemblance of tubercle in its elements to lymphatic tissue, and by its tendency to undergo the caseous degeneration. The granulations of glanders and syphilis also approach each other in their histological elements, aspect, and evolution.)

Thus, then, the question of the specific anatomical nature of tubercle ought to be resolved in a negative sense. The specific globule exists not, and the other characters observed in the histologic evolution are not less insufficient. The granulations of glanders, of syphilis, and of tubercle, present themselves as three species of the same genus, and the two first being inoculable, we are asked if the third would not be. The experimentation has responded. In a work soon to be issued, we try to establish the specific nature of tubercle.

Thus, according to M. Villemin, tubercle will be specific respecting its cause and respecting its nature, but not in its products. These are very different from the inflammatory products in their period of termination, and take on sooner the caseous form; on the other hand, they approach the products of glanders and syphilis, both affections being essentially specific and communicable.

Prof. Sangalli, of Pavia, remarked to the effect that he also assimilated the gray granulations to caseous pneumonia. He found in both the same elements, they succeed in the same order; but that which struck him principally, was the affinity between tuberculosis and inflammation. He attributes the production of granulations or the infiltration of tubercle as due to the action of one stimulus, which manifests itself at first by hyperæmia. Besides, he would observe that tubercular affections can assume different forms, according to the organ attacked; thus, in the uterus, they can give origin to new organic fibres. Here, the Doctor presented a lengthy statistical table, which we omit.

Prof. Crocq, of Bruxelles, remarked, that we find but one type to which we can refer with precision, the cells of gray granulation, it is found in the lymph cells, the lymphatic ganglions, the white blood corpuscles, and in those of mucus which are also like pus. That which constitutes the granulations of gray tuberculization is, then, leucocytes; that which distinguishes them from this is, above all, the absence of intercellular substance. These leucocytes are small because they are not bathed in any liquid. They have but a single nucleus. These leucocytes recognize as points of departure, the cells of the connective tissue, and also the epithelium. In the first instance, the granulations which are formed are surrounded by a shell of connective tissue, which concurs in giving them consistence and elasticity. These facts, being weighed, throw upon the development of tubercle a general *coup d'œil*, which may enable us to comprehend its relations with other pathological acts of the economy.

When one examines an organ in which tuberculization is being developed, one sees points or spots of considerable vascularity. Sometimes, the centres of these points are already consistent and elastic. These points pass, by insensible transition, into tubercular granulizations which are always surrounded by well developed vascular zones. A microscopic examination, brings to view numerous and voluminous vessels, which ramify on the surface, and, by position, seem to penetrate the interior of the tubercular mass.

Injection, infiltration, gelatiniform while gray, etc., these are the phenomena observed at the seat of tuberculization, and these coincide with those which preside in inflammation, so that one cannot well describe one without the other. Vascularization and repletion of tissues are at first equally seen in both cases. In inflammation, also, the cellular elements of connective tissues become engorged, swollen, and obscure, and at last give birth to new generations of cells, similar to leucocytes. These new cells have four destinations:

1st. They are destroyed and the material reabsorbed.

2d. They are transformed into cells of new connective tissue.

3d. They are changed into a liquid intercellular substance, and constitute pus.

4th. They are subjected to fatty degeneration, and form masses which may sojourn indefinitely in the tissues and impregnate themselves with calcareous salts.

The only difference that one can signalize between the products of tuberculization and inflammation is the smaller volume of the cells of the former—like the leucocyte—their unique nuclei, the absence of intercellular liquid. In fact, we find in the tubercle an enfeebled energy, a formative force less marked than in inflammatory conditions.

The symptoms of tuberculosis, especially those of the lungs, confound themselves with chronic phlegmasia, as, for example, pneumonia and bronchitis of slow march. Respecting acute tuberculosis, the nature and the functions of the organ, affected generally throughout its extent, expresses the value of the facts we there encounter. One may distinguish after their seats three different kinds of phlegmasia, *viz.*, lobar, lobular, and, finally, vesicular pneumonia, which may be limited to a single infundibulum, or to a small number of them. There are, also, three forms of tuberculization, which we may, after the preceding, consider as recognizing for a point of departure a pneumonia of special form, which we name tubercular.

Does any one say that tubercle may be the result of a general malady, of an alteration of the blood? No analysis has demonstrated the existence of this; one of the strongest arguments for this idea is hereditary transmission. But it is not the tuberculization itself which is transmitted. I have often examined fœtuses and infants of tuberculous women, but have never with those found tubercles. Only the predisposition is inheritable, that is to say, a certain type of internal structure of tissues which may render them accessible to such pathological phenomena.

From these considerations, it results that tubercle is not a special or specific malady, recognizing for its cause a vice of the blood, but an affection of the same order as inflammation, from which it differs but little. The treatment for tuberculization is none other but that for phlegmasia of the same organ—antiphlogistics, revulsives, and appropriate modifications, applied according to the indications.

The opinions of M. Lebert, as shown in the following extract, were very similar to those of M. Crocq, only they are founded upon experiments more recently made upon animals:

M. Lebert remarked as follows:—"These experiments have been made in my laboratory, with the assistance of my excellent chief of the laboratory, M. le Docteur Wyess. Nevertheless, there being some difference of opinion in the interpretation of facts in our experiments, I take upon myself the responsibility of these generalities. Our experiments are to the number of forty-five, not counting a large number not finished.

A first series, of eleven, relates to the transmission of the products of disseminated chronic pneumonia, of chronic adenitis, and to the appearance of tuberculosis and tubercular granulations of the lungs. Two experiments relate to my ancient researches upon pyæmia. Two dogs, in which many injections of pus have been made in the veins, have presented, one, recent granulations in the lungs; the other, in the lungs and liver; both offering the structure of tubercules in these granulations. In nine experiments, the product of expectoration and of caverns have been as in the eleven, first injected under the skin. These animals have succumbed to pyæmia or septæmia, and have not presented the granulations of infection. In the twenty-third experiment, a biliary fistula had been established in a dog, the subject of poison by phosphorus. After one week, the dog commenced to cough, and at the autopsy, there appeared recent pulmonary granulations, having the same character as the products of the first experiments, *viz.*, disseminated pulmonary granulations, etc. The twenty-fifth and thirty-fifth, relate to the transmission of divers morbid products of hypertrophied lymphatic glands, of melanoma of the horse, of fibro-plastic tumors, of cancroid and cancrous matter. The ten last were made with carbon or with mercury, in the jugular veins. These have been introduced, once, directly in the trachea.

"We commence giving the results of these experiments with the last, which are the most simple. The carbon produced little embolons, followed by cellular hyperplasie, little granulations, and even irritation and cell multiplications more extended, alveolare tubular, etc., even extending to the cells of the connective tissue in the interlobular spaces. The mercury produced, besides, a veritable inflammation of the tunics of both veins and arteries, as I had already proved in 1850. Here we find cellular hyperplasie of the external tunic of the artery, under the form of granulations, or, more diffuse, it may extend a distance along the vessels. In a higher degree, the same action can extend, little by little, forming granulations and indurated inflammatory foci in the way of suppuration, followed by "bronchiectasies" and even caverns.

"In inoculating with morbid products, one observes, in one case, a strong local irritation; in others, numberless granulations in different organs without local action. (Here, M. Lebert supposes that an infectant juice, in one case passes through the lymphatics, in another through the veins, arriving at last at the heart, and from thence to the pulmonary capillaries, where it forms foci of obstruction, and from here a part passes into the aorta, and from thence into the cœliac axis, the hepatic and renal arteries.)

"The impossibility of injecting the foci of obstruction by the vessels, proves a mechanical obstruction, but it must have there also an irritating chemical agent, which must make the liquid traverse the obstructed capillaries, and the obstruction itself, as the collateral hyperæmia does not account sufficiently for this cellular hyperæmia, so notable. When the capillaries of the pulmonary cells are obstructed, the collateral fluxion takes place always through the last ramifications of the bronchial arteries, towards the termination of the bronchioles. This fact comes to the support of the opinion that we have published, that the cellular hyperplasie extends from the bronchioles to the pulmonary alveolæ, for these are the bronchioles that receive the most of the nutritive materials. It is very probable that in all these infections the general laws of irritation and of cellular hyperplasie dominate.

"We see different morbid products. Those of chronic pneumonia disseminated, granulations termed tuberculose, lymphatic glands chronically infiltrated, melanose, and carcinoma, provoke granulations of infection very nearly identical; also, that a hyperplastic action may take place, both in the cells of connective tissue and in the epithelium, and that the differences are not governed by age nor by changes, progressive or retrogressive of the tissues. These products of retrogressive cellular metamorphosis, when absorbed, engender new foci of infection, by irradiation and propagation at a distance, and it is thus the infection perpetuates and multiplies. Nothing is more vague to-day, than the definition of tubercle originating in an action essentially inflammatory, and its results, even its minutest products of secondary infection, have granulations in structure identical with those from inflammation of connective tissue, granulations often surrounded by diffused hyperplasie. The tubercle is a product eminently hyperplastic, so that no limitation can separate it from inflammation, and we do not know how to assimilate it to any accidental product, properly so called."

After the reading of the above, M. Hérard took the stand. The first part of his discourse was a resumé of his work on Pulmonary Tuberculosis. He found in the granulations the only characteristic lesion of tubercle. He isolates it from caseous pneumonia, even more than Virchow. After having repulsed the opinions of those who, as Virchow, connect caseous inflammation with scrofula, that he had found it much more rarely connected than granulations and caseous pneumonia.

M. Hérard asked the question, If this pneumonia followed or preceded the granulations? The German school generally suppose that it precedes, that the granulations are caused by the metastasis and generalization of the caseous products, that the affection changes its physiognomy when the granulations appear. M. H. believes to the contrary, to the preexistence of granulations. Often one may find them isolated, without caseous inflammation in any organ; often one may suspicion them by stethoscopic signs.

After this discourse, M. Villemin followed in a conciliatory manner—more conciliatory than profitable. Therefore, we omit this and many other little equestrian efforts at riding hobby-horses possessed of the vicious habit of "stumbling and bolting the course."

M. Mugeot (de Bar sur Aube) explained by the laws of osmosis, the production of tubercle.

M. Empis replied, that M. M. only touched upon the truth; the fact is, colloid substances cannot traverse membranous septa, unless they are acted upon by great pressure. It requires a great pressure to make them leave the vessels and constitute by their deposit the granulations of tubercular infiltrations. This is why all the affections that are apt to cause tuberculosis, such as scarlatina, measles, etc., are those in which one finds a great sanguine pressure, then it can make infiltrations on the surface of serous membranes, as in acute hydrocephalus. The principal indications are, then, to prevent the development of tubercle, and among moderants, he had found the best to be nitrate of potash. In conclusion, M. Mougeot spoke of the good effects that are derived from breathing steam in the many pulmonary maladies.

*Evening Session, August 17th.—Treatment of Tuberculosis.*

M. Gourdin recounted the results (not very satisfactory) that he had obtained in the treatment of tubercle, by the injection of nitrate of silver in the caverns, and by the use internally, of petroleum and phenique acid.

M. Marchal (de Calvi) spoke against offensive treatment in phthisis pulmonalis. He designated by the word offensive, iron, iodine, sulphur, and, perhaps, quinine. He had often seen these remedies, in subjects predisposed or already tuberculous, bring hæmoptysis and hasten the development of new tubercles. He declared the bad effects of iodine in phthisis, and its good effects in scrofula, and explained this last, that it caused hyperæmia in the glands, caused the development of new bloodvessels, which, in their turn, were the agents of reabsorption, thereby relieving the engorged glands. Thus, M. Marchal separates, absolutely, scrofula from tubercle. According to him, no medication can replace a good hygiene, change of climate by sending patients to a well chosen climate. He believed, also, that cancerous affections and other incurable diseases can be greatly benefited by transferring them to climates where such diseases are rare or not known.

M. Auzias (Turenne) cited several cases of phthisis, which had been greatly ameliorated by the use of garlic.

M. Marcovitz (de Bucharest) spoke against considering phthisis as a unity, or as one species. He wished that the facts might be arranged in such manner that one could distinguish the species. Whilst, for some, tuberculosis is a simple inflammatory affection, others consider it as a specific malady, pertaining to the constitution, the temperament, and idiosyncrasy, and others still, as an exit or termination for many diverse diatheses, as arthritis, scrofula, etc. He ranged himself among the latter. He recognized then, many sorts of phthisis that do not proceed from the same source, have not the same character, and do not demand the same treatment. Among these varieties, he signalized:—

1st. Phthisis hemorrhagia, in which one observes a great cardiac susceptibility, such as palpitations, with fever, from slight causes.

2d. Phthisis subacute, generalized.

3d. Phthisis chronic, with moderate fever, with tendency to hemorrhage.

With the first form, sulphur water is injurious, but beneficial for the third. Respecting iodine, it does not act in the same manner topically as in the stomach; given internally, it may act injuriously in certain cases.

M. Lombard supported M. Marchal's views, and, in addition, remarked that elevated plateaux appeared unfavorable to the development of tubercle, because there is less oxygen received in respiration. These facts had been observed in Peru and in Mexico.

M. Haller (of Prague) approved of the reflections of M. Marchal, upon offensive medication in phthisis. He viewed as such, those that fatigue the digestive canal, diminish the appetite, and, in consequence, induce anæmia.

*Session of Monday, August 19th. Anatomy, Pathology, and Physiology of Tubercle continued.*

M. Empis defended his ideas upon granulation. His discourse was simply a resume of his book.

M. Empis has made a great number of inoculations upon animals, and has been able to make granulation appear in rabbits, in employing morbid products of divers character, as the pus of puerperale peritonites, matter from Peyer's glands in typhoid, pus of neumonia, etc.

But although these granulations are similar to those of tubercle, M. Empis does not admit that they constitute this affection with the rabbit, for the granulation of tubercle is a general affection. He had never seen true phthisis developed from the above experiments.

M. Cornil said: The essential and fundamental points in the history of tuberculosis was the development of tubercular granulations that we would continue to call *true tubercle*.

In serous membranes, and particularly in the pia mater, one may see admirably this development in the adventitious sheaths of the vessels and in the lymphatic sac which surrounds them.

It is in this lymphatic sheath, and circumscribed by it, that is effected at the expense of elements of the sheath itself, and at the expense of the adventitious membrane of the vessels, the proliferation of the little cellular centers which constitute the beginning of tubercular granulation. It is always at the bifurcation of the vessels that these phenomena are witnessed.

It is the initial fact—the proliferation of the external membrane of the vessels, that is to say, an inflammation in the sense usually accorded to that term to-day.

Two other facts of general importance come to be added there: 1st, The multiplication of similar elements in the connective tissue of the pia mater which surrounds the affected vessel at this point; and 2d, The coagulation of the blood, the retrogressive metamorphosis of the fibrine and of the globules in the interior of the vessels in which the circulation is interrupted.

There is, then, at the commencement a phenomenon analogous to arteritis upon the little vessels and capillaries, and a coagulation of the blood analogous to that observed in phlebitis of the large veins; the consequences are striking; as the anæmia of certain parts of the brain and of the pia mater, the augmentation of the collateral pressure of the blood in the neighboring parts, the formation of pus in the meshes of the pia mater, etc.

In the brain substance, the tubercular masses can sojourn an unlimited time, and it is the same in the lungs, where the granulations very much more visible with the microscope can exist in a sort of mummyfied state many years in the midst of connective tissue. (Slate colored interstitial pneumonia.)

In the lungs, the process is more complex: at first, the vessels are altered in the same fashion as elsewhere; the septæ of the lungs show, at the expense of capillary centers, a proliferation of small elements, as around the vessels of the pia mater. But, besides, one finds in the interior of the pulmonary *alveola*, and in the interior of the bronchi, large elements, free, spherical, or pavement which fills them, and in time are subjected to granular, fatty degeneration. It is impossible to confound elements which proceed from proliferation of epithelium with those from connective tissue. The first are free and voluminous, they are epithelial cells tumefied. The second are small elements, and are agglutinated with each other by a homogenous and granular matter. The first constitutes tubercular pneumonia, the second granulation. We will not separate these two processes in their etiology; this is why we have adopted (M. Hérard and myself) the words tubercular pneumonia as preferable to all others, but as to the anatomical nature, we cannot confound them, and in this we disagree with M. Villmin.

A Hungarian, Dr. Bakody, of Pesth, in studying this question, had arrived at similar conclusions with M. Cornil. He showed a large number of designs to the members representing each of the phases of the evolution of tubercle. He said that tubercle, in a anatomical sense, was a heteroplastic neoplasme which destroys the matrix of the tissues, and shows itself habitually in a discrete form, may be in the lungs only, may be at the same time in the other organs, it may appear under the form of multiplied granulations, of the size of millet seeds, or under the form of nodosities, of larger size, constituted by the conglomeration of granulations. Under the microscope, it shows an aggregation of cells which are derived from the connective tissue. It developes itself in the connective tissue, it may be in the submucous, interstitial, in the adventitious tunic of the vessels, or enter into the texture of the culs-de-sacs and the framework of the alveola. The tubercles which have originated in the connective interstitial tissue soon pierce the partitions of the alveola, which they irritate, and this irritation extends itself more and more along the bronchia.

In the alveoles, one finds pretty often another form of granulations, which conceal in their center only pavement epithelial cells. But as these cells of epithelium are sometimes vibratile, and as these, in the normal state, are only found in the bronchi of pretty large calibre, one can attribute their presence in the alveoles to an irritation which has extended from the bronchi.

The secondary state of irritation of the pulmonary woof is in proportion to the cellular and detrital aggregation in alveoles and cul-de-sacs. Tubercular granulations can then develop themselves under the influence of the mass of inflammatory products which have issued by proliferation from the epithelium in a state of hyperplasie, and which irritate the tissue of the neighboring alveoles and cul-de-sac.

This is why tubercles develop themselves by preference in the summit of the lungs, where the respiratory movements are relatively less extended, and consequently reject less completely the cellular mass which has accumulated in consequence of inflammatory irritation.

The prolongation of the stagnation of this cellular mass may give impulsion to proliferation of cells characteristic of tubercle in the connective tissue of the alveoles and cul-de-sacs of the adjacent parts.

The alveoles may become filled with masses of epithelium and of pus, without resulting in the tubercular granulations, but it is only when the products of irritation or of inflammation have passed quickly into the state of fatty degeneration which may have been partly absorbed and partly expectorated. But if this does not arrive promptly, or if new masses succeed the first, the connective tissue which environs becomes irritated, and tubercular granulations form as a consequence.

M. Linas combatted the communication of M. Augens, of Turenne, upon the employment of garlics in phthisis. He recalled that, according to Calius Aurelianus, Mead, Rusen, etc., this remedy proved useful in bronchial catarrh, but that in southern countries, where garlic enters largely into the general alimentation, there was found no less of this disease, which proved the inutility of this plant against tubercle.

M. Lombard, of Geneva, renewed the question of the influence of altitude over the development of tuberculosis. He presented tables, where the proportion of oxygen received in each inspiration was calculated according to the height, and foreseeing an objection which might be made, he acknowledged that phthisis became more rare in proportion to the elevation and as one respires less oxygen. The opposite should obtain as one leaves the Equator towards the Pole, where the air is more dense, being colder.

M. Prof. Friedrich, of Heidelberg, returned to the question of the production of tubercle, nearly in accord with Corriel, but he disagreed in one point. He believes that the granulations may be produced at a distance from the vessels; that wherever corpuscles of connective tissue exist, these may divide, and proliferate and constitute little granulations. M. F. has often found upon membranes, for example, the pia mater granulations thus formed at points distant from any vessels.

[*Query.*—How great is the distance between any two or more vessels in the vascular net work of the pia mater? We are not educated up to the point of answering.]

FIRST QUESTION—SECOND PART.—*Of the tuberculization in different countries, and its influence on the general mortality.*

M. le docteur Marmisse (de Bordeaux) presented a paper entitled "Researches upon the statistics of phthisis pulmonalis as a cause of death in the City of Bordeaux." He read some extracts. This brochure is full of documents upon the age, the sex, profession, etc., of the inhabitants who die of phthisis, and upon the time of the year when this mortality is most considerable. The influence of hygienic and social conditions are indicated in his figures. Of 1000 indigents inscribed to the "bureau of charity," 625 died of phthisis, 315 to 1000 are due to the same malady in the wards of the hospital, while, among the rich, 87 to 1000 is the ratio.

M. Sanamea advocated the importance of good hygiene as a prophylactic against tubercle. He said that bad alimentation, lack of air, of light, and of exercise, should figure among the first causes of phthisis.

Then followed a memoir from M. le docteur Homan, of Christiania. This memoir was enriched with tables of statistics, and with a geographical chart, indicating the distribution of phthisis in the Norwegian provinces.

In one place, he states, that if one unites phthisis with other affections, often considered as tuberculosis, as acute hydrocephalus, scrofula, necrosis, caries, etc., it would amount to a total percentage of 162 to 1000.

At Krugero, where he made his personal notes, 112 to 1000 died of phthisis pulmonalis, 39 of scrofula, caries, and white swelling.

If one examines separately each district, it will be seen that the mortality differs, one from the other, in the proportion of 79 to 226 per 1000. In the northern districts, on the sea, it is relatively more feeble, and does not exceed 10 per 100, except in Drontheim, and the City of Bergen, while it exceeds very much the mean average in the southern districts of the seaboard, except in the prefecture of Christiania, where it attains but 11.15 per 100. In the interior districts, the rate is not much elevated, but higher than on the northern seaboard.

Sometimes one may verify between two districts which touch, and where the climate is nearly the same, a considerable difference, which proves that climate influences do not predominate.

The difference in the extension of phthisis in the different Norwegian countries, he attributes to the difference in degree of the presence of syphilis.

After the researches of Mr. Bœck, this malady was first imported towards the second half of the eighteenth century into the districts where tuberculosis is at present most prevalent. Thus, syphilis with the ancestors became tuberculosis with the descendants.

M. Dropsy (de cracovie.) In the country that I inhabit, the air is pure, the soil fertile, the water delicious, and the temperature moderate. The inhabitants are composed mostly of peasants and Jews.

The villagers are nearly without exception healthy and robust. They are not subject to any other diseases than those of an inflammatory or rheumatic character.

The Jews, on the contrary, are nearly all scrofulous, and among them phthisis makes such ravages, particularly with those from 19 to 20 years, that if circumstances do not change, one can safely predict the extermination of this race in two or three generations.

The cause of these differences in the sanitary state of the Jews and villagers is found in the manner of living. A Jew of this country eats nearly nothing; his living costs hardly two sous per day, and this is not composed of substantial viands—seldom meat, consequently these people are emaciated, and in a state of habitual anemia and of deterioration of blood. They marry at the age of 16 or 18, which contributes to their exhaustion.

Thus, it is not the locality nor the climate which enjoy the grand role in the production of phthisis, but the general impairment, it may be, from insufficient alimentation or any other cause which results in the enfeeblement of the vital forces.

As to medication, I will say but little. The garlic recommended by M. Auzias Turenne makes the base of alimentation with the people in question, and I have already stated what ravages phthisis makes among them. I have incontestable success with baths of milk, but that which has succeeded best is general electrisation. I apply positive electricity to the hands and feet, and negative to the top of the head and pit of the stomach. I make use of a constant current of feeble intensity, and without ceasing on account of cough, fever, or hæmoptysis. The results are excellent.

*Monday, August 19th.* FIFTH QUESTION IN THE PRIMITIVE PROGRAMME.—*Of the influence of climates, of races, and of different conditions of life upon menstruation in the different countries.*

M. Louis Mayer (de Berlin) presented 59 tables of statistical researches upon menstruation in Germany, northern and central. This memoire was too large to be read, too rich in facts, considered under aspects the most variable to be susceptible of of analysis, and was disposed of very honorably among the archives of the Congress.

Also, we will not speak of the tables that M. Lendet, of Rouen, furnished upon the subject of menstruation in the City of Rouen—tables that must be read entire, as precious documents, but which do not suffice for conclusions read separately. We limit ourselves in signalizing the opinions given by M. Lendet upon the fecundity of women in Normandy.

After putting aside the working classes—very fecund—M. Lendet saw no great difference in regard to fecundity, between the rich classes and the paupers, between the same classes inhabiting cities or country, with each the number of infants is very meagre in this province.

M. Gustave Lagneau presented 15,948 observations collected from nearly all countries, which we omit.

M. Jaulin (Paris) in his turn, read a paper, from which we give a résumé.

The solution of the diverse questions which make the subject of this memoir cannot be resolved but by statistics. These statistics embrace a large number of facts, among these, we have:

1st, Influence of climate upon menstruation. In grouping a great number of practical statistics upon the epoque of the first menstruation, I reunited in total 16,517 observations. The ensemble of these documents, relative to people very different, has permitted me to divide into three zones the countries composed in this study. The first zone—temperate—is circumscribed between the 33d and 54th deg. of latitude north. The second—torrid—is comprised between the 33d deg. and the Equator. The third extends from the 54th deg. towards the Pole.

Temperate zone. The tables of this zone are constituted by 16 statistics, including 10,080 parts. The highest of the total of ages is 1,824, and corresponds with the fifteenth year. The fourteenth give 1,114, and the sixteenth, which approaches most nearly 1,562. It is then towards the fifteenth year, in our estimates, that menstruation appear most frequent.

Torrid zone. The tables pertaining to this zone comprehend 1,734 observations; the highest figures, 407, correspond to the twelfth year, notwithstanding that of the thirteenth year (381) approaches it very nearly. There exists, then, a difference of more than two years between the establishment of puberty in our climate and the torrid zone, one is misinformed to invoque precocity of marriage in India as causes of hasty menstruation, for marriage is not consummated until puberty is established.

Frigid zone. These observations embrace 4,713 facts. The highest figures correspond to the fifteenth and sixteenth years, 872 and 874, nearly equal. It is nearly a year later than in the temperate zones.

The results furnished by the examination of the three zones that I have traced, leaves no doubt then regarding the action of climates upon the time of puberty.

Influence of race upon menstruation. These influences may be demonstrated by comparing pure types of the principal races. As for the negroes, we only possess the statistics of Robertson, comprising 89 facts. The mean age is fourteen years and ten months—a little higher figure than in the climate of India.

*Wednesday, August 21st.* SECOND QUESTION OF THE PRIMITIVE PROGRAMME.—*Of the accidents which cause death after surgical operations.*

Extracts from M. Bourgade (of Clermont-Ferrand.) Three important facts dominate all the history of accidents which lead to death after surgical operations.

1st, One does not generally observe the accidents in the country, while they are frequent in cities, and above all in the hospitals and ambulances.

2d, One does not see them follow but rarely the use of caustics. Very frequently, on the contrary, after the use of cutting instruments.

3d, Once developed, they are nearly always mortal.

Prophylaxis. This rests principally upon recognizing the causes. The causes we must search. Now the etiology of these grave complications have been elucidated by what has been said above.

1st, By the innocuousness nearly absolute in the operations practised in the country. 2d, The habitual freedom from complications following the use of caustics, even in places the most unfavorable, as in hospitals. 3d, The frequency of grave accidents following the use of the bistoury.

There is, then, in the midst of all human agglomerations a cause which exercises a grievous influence upon the cure of wounds.

This cause must be found in the production of a miasm or ferment, which is developed in these conditions, and which exercises its deleterious influence, not only upon the organism, but principally upon the wound itself. If this action is not essentially local, why are those operated upon by caustic exempt from these complications, and why do they supervene nearly exclusively after the employment of cutting instruments, which leaves naked a denuded surface, exposed to all the exterior agents?

There is, then, a deleterious local action; it is necessary "par consequent" to strive to subtract it from the wound.

It is because of these dangers that surgeons have wished to limit the use of cutting instruments, substituting these for caustics, or other means of dividing tissues.

But, after all, the bistoury will always be the surgical instrument "par excellence."

It is necessary, then, to conserve its usage, but endeavor to weaken upon the wounds it produces, the deleterious action of morbid agents.

This problem I will solve, and render wounds made by cutting instruments as inoffensive as those made by caustics.

I believe that I have resolved it by the employment of perchloride of iron applied to the wound immediately after the operation. Here is a description of the proceeding employed by me: When I have terminated the operation, and have applied the ligatures, I lave and wipe the wound with the greatest care, and when the flow of blood is well arrested, I cover the entire surface of the wound with pledgets of charpie, soaked in a solution of perchloride of iron of 30° purity. It is necessary that all parts of the wound, even the most anfractuous, as the bone, the muscles, the vessels, and cellular tissue, should be subjected to the action of the liquor chlori-ferrique. The whole is recovered by a mass of moistened charpie.

The perchloride of iron combines then intimately with the tissues, and thus forms a covering which is solid and adherent over the wound, a species of plastic cuirasse, which, at the same time, coagulates and forms an eschar—for, at 30°, the solution of Pruvez is moderately caustic—which acquires hardness and offers resistance, and which does not commence to detach itself under the influence of suppuration before the sixth or eighth day, and sometimes not before the tenth.

There is, then, in the first dressing something which seems to possess, at the same time, the power of occlusion and of cauterization, and unites the advantages.

I never make traction upon the charpie which is adherent to the wound; I leave it to detach itself under the influence of suppuration, aided sometimes by lavement. In falling, it discloses a duskish surface, covered with a thin escharotic bed, which detaches itself in its turn, gradually, and brings to view a surface, rose-colored and healthy, of a beautiful aspect, and already covered with fleshy germs in the way of organization.

The dressings are then made with aromatic wine. The wound furnishes pus of a healthy nature and not abundant, which marches gradually, and without im-pediment, towards cure.

Sometimes it is possible, after the fall of the escharotic bed, to bring the soft parts together and obtain rapid secondary reunion.

During the treatment, and from the commencement, the patients remain in good condition; they suffer little, and have but little traumatic fever, and it does not prevent appetite or sleep.

A somewhat sharp pain follows the application of the perchloride of iron, but, at the end of a few minutes, it diminishes notably, and becomes quite supportable. It is never prolonged beyond a few hours, and confounds itself with the ordinary pains of the operation. It, therefore, does not constitute a contraindication.

One will comprehend that this method is not applicable to immediate reunion of wounds, notwithstanding it allies itself very well with attempts at partial reunion, the best results that one could hope to obtain in hospital practice. In the last endeavor, this method seems to secure success. Since five years, I have employed this method in a general manner at the Hotel Dieu, of Clermont, in all operations that seemed important enough to lead to grave complications. I have applied it in 95 operations, which have all succeeded. The accidents which I have endeavored to prevent by this method are, more especially, purulent infection, phlebitis, angeio-leucitis, osteomyelitis, and consecutive hemorrhage.

The perchloride of iron appears to me to act in these cases as a light cautery to the bleeding surfaces, and exercises a strong coagulating action as far as the interior of the veins. These results are obliterating adhesive phlebitis, which prevents suppurative phlebitis, and opposes the absorption of all morbid matter, all elements dissociated from pus, etc., etc.

M. Barbosa (Portugal) presented a statistical record of the operations practised in St. Joseph Hospital, of Lisbon. He says the greatest mortality corresponds with spring-time, next winter, then summer, last autumn. Among the causes of death, he places first purulent infection, 44 per 100. Afterwards, come erysipelas, 18.6 per cent., drunkenness, exhaustion, etc. In 13 resections, there was but 1 death; 28 lithotomy, 10 deaths; 34 hernia strangulated, 20 deaths; 19 ligatures of arteries, 4 deaths; 19 amputations of the penis, 3 deaths; 407 extirpations of tumors, (diverse,) 16 deaths, etc. He plumed himself on the results, and attributed this good fortune to hygenic conditions, such as good ventilation, frequent washing, and besides in the method of operating and mode of dressing the wounds. He employs, in preference, the circular method, and saws the bone as high as possible. He removes all the clots, and makes methodical compression to the stump, commencing at the upper end of the stump, and from thence toward the wound. A stream of water is directed upon the wound, the angle of the latter being placed below, in order to facilitate the discharge of fluid. The dressings are completed by the aid of tr. camph. and alcohol in excess—an ancient practice in Portugal. The patients are made to drink port wine, and are carried into the garden daily.

M. Gosslin made some remarks in support of the importance of good hygienic and sanitary regulations in the treatment of surgical operations.

M. Gosslin here presented an immense number of statistics of causes of death in his hospital—the Pitié—the burden of which are erysipelas, purulent infections, etc., all of which will appear in detail in the transactions. His method of treatment is as follows:

1st. "I put my patients for operation in the largest halls, where I do not allow erysipelatous patients to remain.

2d. "Always when there is no urgency, I leave the patient time to consider the operation, and to become accustomed to the idea by designating a day, and I seek to dissipate his inquietude respecting the results."

During, I take care to suppress pain altogether by complete anæsthesie, and take much pains in tying the arteries.

After, I endeavor in the first dressings to avoid giving pain by not placing around the stump any circular bandage that would necessitate lifting it. I do not try to approach the borders of the wound, that I cover with a compress, wet with cold or warm water, according to the season. I do not add alcohol, which would add to the pain. I give them as much aliment as possible, and to their taste—wine, and sometimes brandy or rum. I avoid, as much as possible, everything that would give pain, bodily or mentally.

Here are some results:—"I have made 19 amputations of the thigh, 22 of the leg, 4 of the arm, 3 fore-arm—in all 48; of which, 29 were cured, 19 died, which gives a mortality of 39 per 100. Of the 19 deaths, 10 alone were caused by purulent infection. I had, besides, 9 others affected with purulent infection that had not been operated upon."

These figures would not make a very good show with us in the United States, but M. Gosslin prides himself upon the results, and attributes these to the great care bestowed upon the general condition of the patient before and after operating.

If the general state of the patient has this importance in varying success, his habitual state should not be neglected in making the prognosis.

M. Verneuil insisted upon this part of the question. He signalized the influence of a latent diathesis, which may not manifest itself by any symptom, but may be revealed at any instant by an exciting cause. He continued: One knows that lithotomy and lithotrity are grave when the kidneys or bladder are the seats of morbid changes. That tracheotomy is much more benign when practiced for the extraction of foreign bodies than in the diphtheritic condition. That the amputation of a leg is very serious, when covered with varices, superficial or profound. That the prognosis of the amputation of the breast is much more serious for cancer than for adenoid tumor. We commence to know that the most trifling operation may cause death with a person having diabetes. M. Chevers has taught that latent affections of the kidney often explain the death, after operations the most diverse, and of little gravity. But how much of the unknown remains to disengage, and how many contradictions still exist. As the influence of drunkenness, of miasm, of acclimation in the halls of hospitals before the operation, of the period of menstruation, lactation, gestation, etc., upon the results of an operation or traumatic injury. I believe that erysipelas is most frequent in arthritic and herpetic diatheses. I do not believe, as is generally supposed, that amputations and resections are more grave in healthy subjects than with those debilitated with chronic lesions. I propose to devote the better part of my scientific activity and my practical experience, to prove that the general state, ancient or recent, diathetic, hereditary, or acquired, dominates over the prognosis and results of surgical operations, and constitutes the richest source of indications and contra-indications for operations.

M. Labat seemed to attach less importance to the general state of the constitution and diathesis. All his attention seemed to be directed to the wound itself, the method of operating, and dressings. He regards the blood and, perhaps, other liquids that the tissues may have imbibed before the operation, as foreign substances, which can never serve in the reparation, but, on the other hand, corrupt by contact with air, and thus become a source of danger. His method is, then, to remove these, as much as possible, from the surface of the wound, while he takes care to conserve the plastic exudation provoked by the operation, and which furnishes a formative blastema for the elements of the new tissues.

We see that M. le docteur Labat has adopted the views of Robin upon the formation of tissues from a preëxisting blastema. Here are the practical conclusions that he formalized before the Congress:

1st. Do not seek union "by first intention," only when the wound is shallow and the texture of the tissues uniform, when the opposed surfaces can be kept in contact deeply as well as superficially, when the tissues have not been too profoundly contused.

2d. In operations, dispose the flaps in such manner that the flow of liquids can take place with facility, and that they rest easy, one upon the other.

3d. Avoid, with care, all conditions that can lead to the alteration of products, or the retention of the altered products in the vicinage of the open mouths of veins.

4th. Favor the flow of liquids, by establishing drains.

5th. Never neglect a counter opening, when necessary, from the first.

6th. Avoid everything of an irritating nature, particularly in regions abounding with lymphatics.

7th. In wounds which are lacerated or contused and not united, prevent the retention of liquids at the bottom of the anfractuosities, by filling these cavities with charpie.

8th. Leave the member as immovable as possible, and avoid painful dressings.

9th. Abstain, absolutely, from lotions of pure water upon the wound; on the contrary, alcohol and water prevents the alteration of organic matter, and, in this way, renders considerable service.

10th. As long as one fears the supervention of purulent absorption, give ergotine in doses of 2 or 3 grammes the first day, and continue the days following, if necessary.

M. Mazzoni, a distinguished professor from Rome, spoke of the utility of separating the medical and surgical wards. (Unnecessary advice for most countries.) He said that erysipelas, purulent infection, and phlebitis were rare complications in Italy, but that traumatic pernicious fever was very common, and if quinine was not resorted to promptly, that the patients almost invariably died. And again; that puerperal fever, as an epidemic, was unknown in the hospitals of accouchement in Italy.

Prof. Palasciano, one of the Vice-Presidents of the Congress, had collected the statistics from the Maternité Hospital, of Naples, during thirty years, and had shown the complete absence of puerperal fever, although situated in the hospital for incurables, with twelve hundred beds, and where the most elementary hygeine is neglected. He continued:—"What is the cause of these important results? The response is this:—In the hospitals for accouchements and operations, patients are not received who can possibly injure the atmosphere, as those with typhus, typhoid, or other forms of fever; and those with tuberculosis are always separated from all others, and placed in wards distinct and distant.

M. Chauveau has succeeded, recently, in producing the natural vaccine, by inhalation of the contagious matter. The cause of the accidents which complicate surgical operations, is it not sometimes of this nature? In Italy, the surgical patients are cared for as scrupulously as parturient women.

Here followed some boasting between M. Marjolin, of Paris, and M. Meric, of London, about the condition (sanitary and otherwise) of the respective hospitals in Paris and London, which we omit.

Mr. Meric, of London, remarked that, in order to avoid consecutive hemorrhage, he left the stumps open from half to three-quarters of an hour after amputation. He asked M. Verneuil if he had remarked that amputations were much more grave when made upon lesions from railroad injury? For his part, he did not want to appear ridiculous, but he had lost nearly all his patients, and oftenest by gangrene of the stump.

M. le Docteur Bole (of Castel Sanasin) was inscribed for the next talk, and recounted six amputations, one of the thigh, and five of the leg, without losing any. He ascribes his wonderful success to the fact that he always made immediate union, and dressed with perforated linen covered with cerate de Galiem; and that he avoided frequent dressings, leaving the stump without touching many days. M. Bole supposes that similar treatment, if adopted in Paris, would be followed by very satisfactory results; but he forgot that in Paris immediate union is not sought for in hospitals, as the attempts have usually been followed by formidable accidents.

*Wednesday, 23d August.*—THIRD QUESTION OF THE PRIMITIVE PROGRAMME.—*Is it possible to propose to t. e Governments some efficacious measures for restraining the propagation of venereal maladies.*

The first paper on this subject was in the form of a letter, by M. Wleminckx, (Bruxelles,) which was read, in his absence, by M. Crocq, from which we give the following extracts:

"The regulations of prostitution in the city of Bruxelles are the best, or at least the most complete, that I know of. The base of the rules are the following:

"Repeated visits (every three days) to all the women enregistered as prostitutes. Punishment of those who avoid the visits, and recompense to those who never fail to present themselves—this consists in the restitution, at the fifth visit, of the sums paid by them previously, for admission. Those presenting the least suspicion of disease of the genitals are sent to the hospital. Physicians are prohibited visiting or treating prostitutes at their domiciles.

"By these measures, we have seen, in a very short time, the number of cases decrease very much in our hospitals, civil and military, and those of a secondary or tertiary character have almost disappeared. To these general measures I have added one more, specially applicable to the army:—I prescribe that each man entered as syphilitic in the hospitals shall be interrogated upon the origin of the disease, however trifling it may be, and the place where he may have contracted it, and the woman who has contaminated him. I recompense those who make a correct statement. The woman accused is immediately arrested and placed in hospital. The result has been, that syphilis has been nearly extinguished in Belgium."

M. Crocq supported, verbally, the facts communicated in the above letter, and stated, in addition, that gonorrhœa had also become much diminished, but in a degree much less than syphilis. According to our laws, prostitution is exclusively under the control of the corporations of villages and cities. The large cities have regulated it effectually, but there are still "communes" which have not followed this example, and are situated at the very gates of our large cities. They become, thus, the refuge of clandestine prostitution, and the indestructible centres of syphilis. The International Congress of Public Hygiene, held at Bruxelles in 1852, was occupied with this state of things, and proposed measures, some legislative and general, others of a local character. These demanded, amongst other things:

"The interdiction of all prostitution not under the *legal rules;* the personal responsibility of those who kept houses of debauch; the interdiction of prostitution with minors up to a certain age, and the confinement of those who proved delinquent in 'houses of reform;' severe penalties for those who are culpable of facilitating the debauchment of minors; a special tutelage for those children whose parents or guardians favor their prostitution or corruption."

These measures were recommended to be the objects of a special law, respecting the police of prostitutes, etc.

"The intervention of the State is necessary in two points of view, if we wish to attain the end to which we aspire:—First, to indicate to the cities and 'communes' the obligations that they should fulfil, relative to prostitution. Second, to support, at public charge, prostitutes undergoing treatment for venereal disease."

M. Prof. Jeannel presented a lengthy memoir upon prostitution and syphilis. He would impose upon public women, and especially matrons who employ them, the payments of the expenses of treatment in hospital. He proposed to submit all the immoral population to the authority of the "physicians of the epidemics," and to the inspector-general of the sanitary service, who should regulate the number of visits, inspections, etc. He proposed equally to make submit all sailors in ports, either at their arrival or departure, to a rigid inspection, for to these latter, syphilis owes its propagation more than from any other source."

M. Rollet, in the name of the Society of Medicine of Lyon, presented a long and important report, from which I make the following extracts:

The application of sanitary visits to all the prostitutes in all countries, is one of the most important international hygienic measures that one might propose to the different governments. Sanitary visits to men are useful under all circumstances, where there is reason to believe there is risk of propagation of venereal maladies; but these visits are not practicable by the administration, only with those under the immediate control of the authorities.

Without prescribing, in an express manner, to the keepers of houses of ill-fame, the means to be made use of in order to exclude unclean men, he recommended the employment of all practicable means of examination; and all necessary assistance for the girls in preserving themselves from unclean contact, and in no case to be constrained to come in relation with a sick man. The execution of these measures will rest with the mistress of the house, for which she is responsible under penalty, and she shall be subjected to the infliction of damage, when the percentage of disease in her house exeeeds a certain mean average.

The marines and soldiers, whom statistics show to be the most active propagators of venereal affections, should be subjected not only to periodical visits, but at each displacement, embarkment, debarkment, change of garrison, departure on holiday, on returning to the corps. Sailors of merchant vessels should not be permitted to land without certificates of health. The same rules should be applied to sailors of all those nations who may have given in their adherence to the recommendations of the International Sanitary Committee of 1853. These visits should be imposed upon all prisoners, and those arrested for vagabondage.

M. Mougeot believed that clandestine prostitution was the fruitful source of venereal disease, and proposed to drive the unfortunates into the public houses of ill-fame. For those who submit *en carte*, he proposes to render the proprietor legally responsible for the consequences. Respecting personal preservation, he recommends phénique acid or amylique ether, as destructive of the virus, which, according to him is a parasite, with different species for each variety of venereal disease.

M. Auzias Turenne pretended that the only efficacious measure against the propagation of syphilis is syphilization, artificial and methodical. According to him, repeated inoculation destroys all susceptibility. A public woman thus syphilized, and carrying a certificate to that effect from the "syphilisateur," can no more take the disease nor give it. Thus, all the morbid centres would be extinguished in a short time, if such measures became general, and the problem would be solved. He declared, as inefficacious, all other means, administrative or personal. A woman who had been syphilitic, under certain circumstances could—as of an excitation too prolonged—be seized with a relapse of discharges, which, to all appearance, might be simple, yet would be able to communicate syphilis. Thus, the "visits" are rendered null in results, for the prostitutes having, nearly all, a hypersecretion from the genitals, a simple "flow" cannot always be distinguished from the virulent. With those who have been inoculated a sufficient number of times, the secretions can no longer communicate disease. M. Auzias Turenne finally appealed to the Congress to render to syphilization the place which it deserved as a prophylactic and hygienic measure.

M. Ricord presided. "I will make," said he, "but a single observation to M. Auzias Turenne, that which I have always made to him:—If he considers syphilization so efficacious, if he is convinced, let him prove it to us; let him experiment upon himself."

*M. Auzias Turenne.*—"I am ready to present all my observations, those ought to suffice. Scientific questions should remain impersonal."

*M. Ricord.*—"I do not wish to reflect upon M. Turenne. There was nothing personal, in fact, in my question. I said to him, furnish yourself, upon yourself, the proof of your conviction. You have experimented much upon others; you have been able to see if these experiments were innocent; you said they were; prove then, by testimony indisputable, and I will be ready to believe; until then, I say that if you hesitate, you have not the certitude that you proclaim."

President Bouillaud interposed, and said that he had always appeared in defence of scientific progress, but that M. Ricord had the right to ask of M. Auzias Turenne an experience upon himself, that would be the proof of solid conviction; proof that Desgenette gave in relation to the plague; Charvin in yellow fever; the anti-contagionists of 1832, in relation to cholera; M. Ricord, in relation to secondary accidents in syphilis. All have inoculated themselves with that which they thought not to be transmissible, and in view of these acts of courage, one cannot comprehend the refusal of M. Auzias Turenne.

*M. Auzias Turenne.*—"I await scientific objections."

M. Jeannel, for his part, considered a single experiment, personal or not, as offering less proof than a series of anterior observations. The matter of greatest importance is, that M. Auzias Turenne shall produce a large number of facts.

*M. Bouillaud.*—"I wish to say, that as far as I am concerned, I am not opposed to any facts or experiences."

*M. Ricord.*—"I demand not only new facts, but proof of personal conviction."

Here, the general attention was distracted by an unexpected incident:—M. Villemin rose, and, in a voice of thunder, exclaimed:—"See me! I am a doctor of medicine; I am syhpilized, and am very well."

*M. Ricord.*—"Very well then, let M. Auzias Turenne follow your example."

*M. Villemin.*—"I have renounced marriage, he has not."

*M. Ricord.*—"And why have you renounced marriage, if syphilization has the advantages you suppose? Now you are incapable of taking syphilis, or of transmitting it to your wife or, hereditarily, to your infants, and still you renounce marriage. You are a phœnix, in your eyes, for the woman that you would take. This theory of syphilization reposes upon false principles; it implies the unity of the syphilitic virus. I believed a long time in this unity, but observation has convinced me to the contrary. M. Ricord here gave a history of his labors and opinions. He endeavored to show that he had taken the first step towards the truth, by the distinction of two chancres, of which one was the point of departure for constitutional disease, while the other leads to results purely local. From the distinction of two chancres followed the distinction of two viruses. M. Ricord recalled the incident of having informed a young student in medicine that, in inoculating himself with soft chancre, he would take vérole if he took anything, and it took place. If this last is reproduced, we will be able to conclude that the difference pertains not only to the ground, but to the seed also. In a word, M. Ricord showed how he had gradually changed his opinion—constrained by the facts—until he had become a complete dualist, now, to believe in syphilization, one must first become a unicist.

"Our confrére who is syphilized, is he enough convinced to submit to inoculation from the virus of a hard chancre?"

To this, M. Villemin made no reply.

*M. Ricord.*—"I have never inoculated but myself, or the patient himself, with the accidents which pertained to him. This exclusive method of auto-inoculation has caused one of my most important errors. I have inoculated secondary accidents upon the vérolés and I accomplished nothing, for a vérolé can no longer take the vérole. If I had made inoculations from the sick to a well man, I would have been able to discover the truth. But I will never permit myself to make such experiences; when others make them, I will be convinced."

*M. Auzias Turenne.*—"I will restrain myself within the scientific limits of the question:—Exists there one or two kinds of virus? I will cite one fact. I inoculated a person infected with cancer, with the virus of soft chancre; towards the fourth inoculation upon both arms, a chancre, thus produced, was indurated, engorged. I believed, at first, that this was a manifestation of the cancerous diathesis, but a roseole that supervened at the end of a fortnight, and, a little later, the most evident secondary accidents, left no room for doubt. Thus, the pus from a soft chancre had produced a hard chancre and constitutional syphilis. Here are the facts inversed, when we have no soft chancres from which to obtain pus, we fabricate it. It suffices to cover a portion of mucous tissue with a solution of sylphium egrenicum, and three or four days after, in inoculating the pus which it secretes, one produces a soft chancre, indefinitely transmissible. Formerly, Bœck wrote to me often, asking for virus from soft chancre, which he lacked at Christiana during the winter; but now he fabricates it when he wishes. I go still farther:—Suppose the existence of two viruses, is that a reason why transmission may be impossible. The 'small-pox' and 'vaccine,' are they not also different, at the same time one may be prophylactic of the other."

*M. Ricord.*—"I have said from the first, that if soft chancre preserves from hard chancre, it acts in relation to itself, as vaccine to variola, which is an accident, local, preserving from an affection of a general character. As for the transformation of chancres from one to the other, I do not believe it possible."

Adjourned for the Grand Banquet.

